# Bioprinting and Differentiation of Adipose-Derived Stromal Cell Spheroids for a 3D Breast Cancer-Adipose Tissue Model

**DOI:** 10.3390/cells10040803

**Published:** 2021-04-03

**Authors:** Hannes Horder, Mar Guaza Lasheras, Nadine Grummel, Ali Nadernezhad, Johannes Herbig, Süleyman Ergün, Jörg Teßmar, Jürgen Groll, Ben Fabry, Petra Bauer-Kreisel, Torsten Blunk

**Affiliations:** 1 Department of Trauma, Hand, Plastic and Reconstructive Surgery, University of Würzburg, 97080 Würzburg, Germany; Horder_H@ukw.de (H.H.); rbj291@alumni.ku.dk (M.G.L.); Bauer_P1@ukw.de (P.B.-K.); 2 Department of Physics, Friedrich-Alexander University Erlangen-Nürnberg, 91052 Erlangen, Germany; nadine.grummel@fau.de (N.G.); bfabry@biomed.uni-erlangen.de (B.F.); 3 Chair for Functional Materials in Medicine and Dentistry, Bavarian Polymer Institute, University of Würzburg, 97080 Würzburg, Germany; ali.nadernezhad@fmz.uni-wuerzburg.de (A.N.); j-herbig@web.de (J.H.); joerg.tessmar@fmz.uni-wuerzburg.de (J.T.); juergen.groll@fmz.uni-wuerzburg.de (J.G.); 4 Department of Medicine, Institute of Anatomy and Cell Biology, University of Würzburg, 97070 Würzburg, Germany; sueleyman.erguen@uni-wuerzburg.de

**Keywords:** adipose-derived stromal cells, adipose tissue, bioprinting, breast cancer model, extracellular matrix, hyaluronic acid, spheroids

## Abstract

Biofabrication, including printing technologies, has emerged as a powerful approach to the design of disease models, such as in cancer research. In breast cancer, adipose tissue has been acknowledged as an important part of the tumor microenvironment favoring tumor progression. Therefore, in this study, a 3D-printed breast cancer model for facilitating investigations into cancer cell-adipocyte interaction was developed. First, we focused on the printability of human adipose-derived stromal cell (ASC) spheroids in an extrusion-based bioprinting setup and the adipogenic differentiation within printed spheroids into adipose microtissues. The printing process was optimized in terms of spheroid viability and homogeneous spheroid distribution in a hyaluronic acid-based bioink. Adipogenic differentiation after printing was demonstrated by lipid accumulation, expression of adipogenic marker genes, and an adipogenic ECM profile. Subsequently, a breast cancer cell (MDA-MB-231) compartment was printed onto the adipose tissue constructs. After nine days of co-culture, we observed a cancer cell-induced reduction of the lipid content and a remodeling of the ECM within the adipose tissues, with increased fibronectin, collagen I and collagen VI expression. Together, our data demonstrate that 3D-printed breast cancer-adipose tissue models can recapitulate important aspects of the complex cell–cell and cell–matrix interplay within the tumor-stroma microenvironment.

## 1. Introduction

Human tissues are organized in a complex three-dimensional architecture, with cells receiving biochemical and mechanical cues from neighboring cells and the extracellular matrix (ECM) that define cell function [1,2]. It is widely accepted that compared to standard 2D tissue culture conditions, 3D culture better mimics the in vivo environment, and accordingly, 3D cancer models can provide more physiologically relevant readouts, for example, concerning the identification of biomarkers or drug response [3,4,5].

In recent years, the research field of biofabrication, including printing technologies, has emerged as a powerful approach to the generation of engineered living tissues not only for regenerative medicine but also for the design of disease models [6,7,8,9]. 3D bioprinting technologies enable the fabrication of complex 3D cellular constructs allowing the deposition of cells in a defined spatial arrangement [7,10,11]. In extrusion-based bioprinting, characterized by sequential deposition of cell-laden bioink layers, usually, individual cells are printed in hydrogel-based inks. More recently, the use of multicellular aggregates, such as spheroids, has been proposed as an additional approach [7,12]. In spheroids, cells’ self-assembly has been shown to create an intrinsic microenvironment that allows cells to interact with each other within their secreted ECM [2]. Spheroids can be regarded as microtissues that were originally suggested as building blocks for regenerative tissue engineering approaches [6,13,14], and in this context, extrusion-based bioprinting of spheroids was demonstrated [15,16,17]. 3D printing technologies, however, can also be used to include such microtissues in advanced 3D constructs with complex predesigned configurations and spatial control of different cell types to fabricate more physiological in vitro models, such as in cancer research. Bioprinted cancer models could offer the possibility to better mimic the complex interaction of cancer cells with their microenvironment, whose contribution to tumor progression is now widely recognized [18,19,20,21,22]. Malignant tumor cells were shown to activate the local stroma leading to altered signaling by variations in the ECM and cytokine secretion of stromal cells, which by means of a reciprocal crosstalk drives proliferation and migration/invasion of tumor cells [23].

Many tumors develop in close proximity to adipose tissue, and a growing body of literature indicates that cancer cells induce adipose tissue dysfunction, which in turn may support the growth of cancer cells and promote cancer aggression [24,25,26]. In breast cancer, adipocytes as major constituents of the mammary fat pad have only recently been recognized as active modulators of the tumor environment favoring cancer progression [24,25]. Thus, there is a growing need to implement 3D adipose models in cancer research to investigate the adipocyte-cancer interaction [18,27,28,29].

To date, 3D adipose tissue constructs have been mainly generated for regenerative applications using classical tissue engineering techniques with ASCs as a versatile, easily accessible cell source [30]. Concerning 3D bioprinting approaches, adipogenic differentiation of printed ASCs has only recently been demonstrated in a few studies [16,31,32,33,34]. In those studies, the adipogenic differentiation of the printed cells was assessed by histological staining of lipid droplets and gene expression analysis of adipogenic marker genes. Functional parameters of the differentiated cells, such as adipose-specific ECM development or secretion of adipokines, have yet not been investigated in printed constructs.

In previous studies, we generated multicellular spheroids derived from human ASCs and differentiated them towards the adipose lineage resulting in microtissues with adipocytes in a native adipose tissue-like ECM. These adipose microtissues were then combined with tailored melt electrowritten scaffolds to generate sheet-like adipose constructs for reconstructive purposes [13,35].

In the present study, we assessed the printability of the ASC spheroids and their ability to differentiate into adipose-like microtissues within the printed constructs, which were further used as the basis for a 3D breast cancer-adipose tissue model. A hyaluronic acid-based hydrogel formulation, which, in a similar form, was previously shown to be suitable for printing suspended single mesenchymal stromal cells [36], was modified to ensure homogeneous distribution of the spheroids and printing parameters were defined to optimize viability. Adipogenic differentiation was demonstrated comprehensively after printing concerning accumulated lipid content, expression of adipogenic marker genes, and ECM profile. As a proof-of-concept, we 3D-printed a co-culture model with ASC spheroids and MDA-MB-231 breast cancer cells and demonstrated tumor cell-induced modulation of the lipid content and the ECM within the adipose microtissues.

## 2. Materials and Methods

### 2.1. ASC Expansion and Spheroid Generation

Human ASCs were obtained from Lonza (Basel, Switzerland; human adipose-derived stem cells). ASCs were expanded in growth medium consisting of Dulbecco’s Modified Eagle’s Medium/Ham’s F-12 (DMEM/F-12; Life Technologies, Carlsbad, CA, USA), supplemented with 10% fetal bovine serum (FBS; Life Technologies), 1% penicillin/streptomycin (100 U/mL penicillin, 0.1 mg/mL streptomycin; Life Technologies), and 3 ng/mL basic fibroblast growth factor (bFGF; BioLegend, London, UK) at 37 °C and 5% CO_2_. The growth medium was changed every other day. At 80–85% confluence, cells were passaged or harvested using 0.25% trypsin-EDTA solution (Life Technologies). Cells at passage 6 were used for subsequent experiments.

For ASC spheroid formation, culture was performed utilizing agarose molds cast by MicroTissues® 3D Petri Dishes® (16 × 16 arrays, Sigma-Aldrich, St. Louis, MO, USA) according to the manufacturer’s instructions (1 agarose mold per well in a 12-well plate, generating 256 spheroids each). A total of 12,800–1,280,000 cells/agarose mold were cultured in basal medium consisting of DMEM/F12 with 10% FBS and 1% penicillin/streptomycin for 2 days, resulting in the assembly of 256 multicellular spheroids per agarose mold (50–5000 cells/spheroid). Images of spheroids in agarose molds were captured utilizing an Olympus IX51 inverted light microscope equipped with an XC30 digital camera (Olympus, Hamburg, Germany), followed by an application of Olympus cellSens™ Dimension Microscope Imaging software to determine spheroid diameters.

### 2.2. Preparation of Hyaluronic Acid-Based Hydrogels

For hydrogel preparation, a 1.6 wt% thiol-modified hyaluronic acid (HA–SH; Glycosil®, Advanced BioMatrix, San Diego, CA, USA, 10 mg/mL in PBS) stock solution was prepared following the manufacturer’s specifications. Another stock solution containing 1 wt% allyl-functionalized poly(glycidol) (P(AGE-co-G); crosslinker, kindly provided by PolyVation BV, Groningen, Netherlands), and 0.1 wt% Irgacure 2959 (photoinitiator, Sigma-Aldrich, St. Louis, MO, USA) was prepared in PBS and sterile-filtered through a 0.2 µm syringe filter. For the final hydrogel formulation, HA–SH stock solution was mixed with the P(AGE-co-G) and I2959 stock solution in a volume ratio of 1:1, resulting in a hydrogel precursor solution with 0.8 wt% HA–SH, 0.5 wt% P(AGE-co-G) and 0.05 wt% I2959. Depending on the experiment, 0.5–1.5 wt% of unmodified high molecular weight hyaluronic acid (hmHA, 1000–2000 kDa, Biosynth Carbosynth, Staad, Switzerland) was added to the formulation to increase viscosity.

### 2.3. Printing Process of ASC Spheroids in Hydrogels

Before the printing process, ASC spheroids (2500 cells per spheroid) were suspended in the hydrogel precursor solution (4800 spheroids/mL) and transferred to the cartridge of a DC 200 dispensing unit (Vieweg, Kranzberg, Germany). Spheroid–hydrogel suspensions were then printed through a 250 µm or 330 µm steel nozzle (Nordson EFD, Westlake, OH, USA) at 1–5 bar and collected in a reaction tube. Subsequently, spheroid–hydrogel suspensions were transferred into glass rings (40 µL per ring, 5 mm diameter, 2 mm height), as it was also done with hydrogels for the cast constructs. Thereby, constructs in the same geometry for both printed and cast groups were generated to enable comparability, as was described previously [36]. Hydrogels were crosslinked using UV radiation (365 nm) for 10 min. Afterward, ASC spheroid-laden constructs were cultured under adipogenic conditions for up to 21 days, and in further experiments, subjected to co-culture with MDA-MB-231 breast cancer cells (see below). Macroscopic images of ASC spheroid-laden hydrogels were captured using an OZL-464 stereo zoom microscope (KERN, Balingen, Germany).

### 2.4. Analysis of Spheroid Sedimentation Behavior

ASC spheroids were generated, and spheroid–hydrogel suspensions were prepared as described above, without the addition of the photoinitiator I2959. Unmodified high molecular HA was added as viscosity enhancer in different concentrations (0.5, 1, 1.5 wt % hmHA), and sedimentation behavior of spheroids was compared to that in suspensions without hmHA. A total volume of 300 µL of the respective spheroid–hydrogel suspensions was transferred into plastic cuvettes (1.5 mL, Brand GmbH, Wertheim, Germany) and placed on a contact angle system (OCA20, DataPhysics Instruments GmbH, Filderstadt, Germany). The camera of the OCA20 was focused on the cuvette window, and images were taken every 2.5 s for 30 min using the SCA20 software (DataPhysics Instruments GmbH, Filderstadt, Germany). For quantitative characterization of spheroid sedimentation, spheroid number in four vertical sectors was determined after 30 min as compared to the initial number of spheroids. A fraction of 25% in each of the four sectors indicates equal distribution.

### 2.5. Determination of Shear Viscosity

Shear viscosity of hydrogel precursors with and without different concentrations of additional hmHA (*w*/*o*, 0.5, 1, 1.5 wt%) was determined using an Anton Paar MCR 702 rheometer (Anton Paar, Graz, Austria) with a 25 mm parallel plate geometry and a gap of 0.3 mm. The shear rate sweeps were performed at 37 °C within a range of 0.1–100 s^−1^. Duration of data acquisition time for the measurement points was set to change in a logarithmic scale between 10 to 2 s for low to high shear rates.

### 2.6. Adipogenic Differentiation within ASC Spheroid-Laden Constructs

After printing or casting, ASC spheroid-laden constructs were cultured in an adipogenic differentiation medium consisting of Preadipocyte Basal Medium-2 (Lonza, Basel, Switzerland) supplemented with 10% FBS, 1% penicillin/streptomycin, and the hormonal inducers insulin (final concentration 1.7 µM; PromoCell, Heidelberg, Germany), dexamethasone (1 µM; Sigma-Aldrich, St. Louis, MO, USA), 3-isobutyl-1-methylxanthine (IBMX, 500 µM; Serva-Electrophoresis, Heidelberg, Germany) and indomethacin (200 µM; Sigma-Aldrich, St. Louis, MO, USA). Constructs were cultured for up to 21 days, with media exchange every other day.

### 2.7. Co-Culture of Differentiated ASCs and MDA-MB-231 Breast Cancer Cells

For co-culture experiments, hydrogels containing MDA-MB-231 breast cancer cells (ATCC, Manassas, VA, USA) were printed on top of the ASC-laden constructs after 21 days of adipogenic culture. MDA-MB-231 breast cancer cells were expanded in growth medium composed of DMEM (1 g/L glucose, Life Technologies) supplemented with 10% FBS, 1% L-glutamine, 1% MEM non-essential amino acids solution, 1% sodium pyruvate, 0.6% HEPES, and 1% penicillin/streptomycin (all Life Technologies). After harvesting, MDA-MB-231 cells at passage 6 were suspended in hyaluronic acid-based hydrogel precursor solution and printed at 1 bar as described above. Before transferring the suspension into the glass rings for UV irradiation (see above), differentiated ASC spheroid-laden hydrogels were put at the bottom inside the glass ring. The hydrogel–cancer cell suspension was then dispensed on top and UV-crosslinked at 365 nm for 10 min. The resulting co-culture hydrogels (bottom: adipogenically differentiated ASC spheroids, top: MDA-MB-231 cells) were cultured for 9 days in maintenance medium consisting of DMEM/F12, 10% fetal bovine serum, 1.7 µM insulin, and 1% penicillin/streptomycin. Samples were harvested at d0 and d9. Double gels with either empty top or bottom compartment (only ASC spheroids at the bottom or only MDA-MB-231 at the top) served as monoculture controls.

### 2.8. Live/Dead Staining

Cell viability within hydrogel constructs was assessed using a live/dead cell staining kit (PromoKine, Heidelberg, Germany). Hydrogels were washed with PBS and incubated in live/dead staining solution (4 × 10^−6^ M ethidium homodimer III (EthD-III), 1 × 10^−6^ M calcein acetoxymethyl ester (calcein-AM)) for 45 min at RT on an orbital shaker. Images were taken using a fluorescence microscope (Olympus IX51/XC30). To determine the influence of different printing parameters on the viability of ASC spheroids, four live/dead images per construct were taken after the printing process. On all images, spheroids with a damaged outer shell and undamaged spheroids were counted, with a total of at least 80 spheroids per construct and three constructs per group.

### 2.9. Quantification of Intracellular Triglyceride and DNA Content

Intracellular lipid accumulation was determined enzymatically using the serum triglyceride determination kit (Sigma-Aldrich, St. Louis, MO, USA). Therefore, spheroid-laden hydrogels were harvested in 0.5% aqueous Thesit solution (0.5% Thesit in H_2_O; Gepepharm, Hennef, Germany) and homogenized with a TissueLyser II (Qiagen, Hilden, Germany) at 25 Hz for 5 min, followed by sonification (Sonopuls; Bandelin electronic, Berlin, Germany). Subsequently, quantification of triglyceride content was carried out according to the manufacturer’s instructions and measured with a spectrofluorometer (Tecan GENios pro; Tecan, Crailsheim, Germany) at 570 nm. Results were normalized to the total amount of DNA in the respective hydrogel lysates. For quantification of the DNA content, hydrogels were harvested in phosphate/saline buffer (50 mM phosphate buffer, 2 mM Na_2_EDTA * 2 H_2_O, 2 M NaCl, pH 7.4; all obtained from Carl Roth, Karlsruhe, Germany) and homogenized as described above. Using the intercalating dye Hoechst 33258 (Polysciences, Warrington, USA), DNA content was determined fluorometrically (Tecan GENios pro; Tecan, Crailsheim, Germany) at an excitation wavelength of 365 nm and an emission wavelength of 458 nm. Salmon sperm DNA served as a standard.

### 2.10. Quantitative Reverse Transcription Polymerase Chain Reaction (qRT–PCR)

For gene expression analysis, spheroid-laden hydrogels were homogenized in TRIzol® reagent (Life Technologies) using a TissueLyser II (Qiagen, Hilden, Germany) at 25 Hz for 5 min, followed by RNA isolation according to the manufacturer’s specifications. cDNA synthesis from total RNA was performed using the ImProm-II reverse transcription system (Promega, Mannheim, Germany). qRT–PCR was carried out using self-designed, intron spanning primer pairs (Table 1). Mesa Green qPCR MasterMix Plus MeteorTaq polymerase (Eurogentec, Seraing, Belgium) was used for detection. The resulting gene expression levels were normalized to the housekeeping gene eukaryotic translation elongation factor 1 alpha (EF1α; self-designed) for each experimental group and time point. The 2^−ΔΔCT^ method [37] was used to determine the x-fold increase in expression levels for each gene, and obtained values were further normalized to the respective day 0 sample values.

### 2.11. Detection of Secreted Adiponectin

Supernatants of ASC spheroid-laden hydrogel cultures were collected on days 2, 9 and 21 of adipogenic differentiation and were stored at −20 °C until further use. Adiponectin concentrations were determined utilizing human adiponectin/Acrp30 DuoSet ELISA (R&D Systems, Minneapolis, MN, USA). Adiponectin levels were normalized to the total DNA content of the respective samples.

### 2.12. Histological and Immunohistochemical Analysis

Spheroid-laden hydrogels were harvested and fixed in 3.7% buffered formalin at 4 °C overnight, embedded in Tissue Tek O.C.T. (Sakura Finetek, Torrance, CA, USA) and incubated in wet chambers at room temperature overnight. Samples were then shock frozen in liquid nitrogen and cryosectioned using a cryostat (6 µm per section; CM 3050S, Leica, Wetzlar, Germany). To histologically assess adipogenesis within differentiated constructs, cryosections were stained for accumulated lipids with Oil Red O solution (3 mg/mL Oil Red O in 60% isopropanol; Sigma-Aldrich, St. Louis, MO, USA). Cross-sections of the co-culture constructs were stained with Mayer’s hemalum (Carl Roth, Karlsruhe, Germany) and eosin (Sigma-Aldrich, St. Louis, MO, USA) (H&E) to visualize the interface between the adipose and breast cancer cell compartment. For immunohistochemical analysis of deposited ECM components within ASC spheroids, antigen retrieval was performed using proteinase K digestion for 10 min at room temperature, followed by three washing steps with PBS. Cryosections were blocked with 1% bovine serum albumin in PBS for 1 h at room temperature and incubated with primary antibodies (anti-collagen I, ab34710, 1:400; anti-collagen IV, ab19808, 1:150; anti-collagen VI, ab6588, 1:200; anti-laminin, ab11575, 1:200 and anti-fibronectin, ab2413, 1:500; all from Abcam, Cambridge, UK) diluted in 1% BSA in PBS. After incubation in a humidified chamber overnight at room temperature, cryosections were washed three times with PBS and incubated with the secondary antibody (goat anti-rabbit Alexa488, ab150077, 1:400, Abcam, Cambridge, UK; diluted in 1% BSA in PBS) in a darkened humidified chamber for 1 h at room temperature. After three additional washing steps in PBS, sections were mounted with DAPI mounting medium ImmunoSelect (Dako, Hamburg, Germany), and images were taken with a fluorescence microscope (Olympus BX51/DP71). For immunohistochemical detection of MDA-MB-231 breast cancer cells within the co-culture constructs, anti-pan cytokeratin AE1/AE3 primary antibody (1:50; ab27988, Abcam, Cambridge, UK) was applied [38,39] along with goat-anti mouse Alexa488 secondary antibody (1:200; 115-545-146, Jackson ImmunoResearch, Ely, UK). Histomorphometric analysis of the fluorescently stained ECM components was performed on 10 images of ASC spheroids per condition. Images were post-processed with Fiji (ImageJ 1.53c) in combination with the BioVoxxel plugin [40] to determine the mean gray value of each staining [41]. Subsequently, the mean gray value of the negative control was subtracted from the mean gray value of the respective staining to obtain the corrected mean intensity.

### 2.13. Statistics

Statistical analysis was performed using OriginPro 2020. Quantitative data are presented as mean ± standard deviation. Each experiment was conducted at least three times with *n* = 3 replicates, if not stated differently. When two groups were compared, statistically significant differences were assessed by an unpaired two-sample Student’s *t*-test. Statistically significant differences between groups with two independent variables were assessed by two-way analysis of variance (ANOVA), significances between groups with three independent variables were assessed by three-way ANOVA, both in conjunction with Bonferroni post hoc analysis. Values of *p* < 0.05 were considered statistically significant and are indicated by *.

## 3. Results and Discussion

### 3.1. Generation of ASC Spheroids

**Figure 1:**
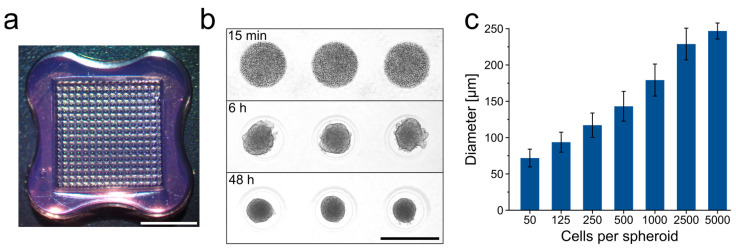
Generation of ASC spheroids in large quantities for 3D bioprinting. (**a**) 3D Petri Dishes^®^ were applied to obtain agarose molds for the large-scale production of ASC spheroids (256 spheroids/mold). Scale bar represents 5 mm. (**b**) Assembly of ASC spheroids in agarose molds after 15 min, 6 h and 48 h (2500 ASCs/spheroid). Scale bar represents 500 µm. (**c**) Average spheroid diameters as a function of the number of ASCs per spheroid. Data are presented as mean ± standard deviation.

For use in 3D bioprinting, the generation of spheroids on a large-scale and in uniform size and shape is required. For this purpose, the spheroids were formed in agarose-based micromolds (Figure 1a), allowing for the fabrication of 256 spheroids per well, i.e., a total of 3072 spheroids per 12-well plate. Spheroids formed within 48 h in a highly controlled manner with a regular round shape and reproducible size (Figure 1b). The size of the microtissues was easily adjustable by using different seeding densities (Figure 1c). Spheroids with 2500 cells and a diameter of 228 µm (± 22 µm) were routinely used in the following printing experiments.

### 3.2. Determination of Processing Parameters for 3D Bioprinting of ASC Spheroids

**Figure 2:**
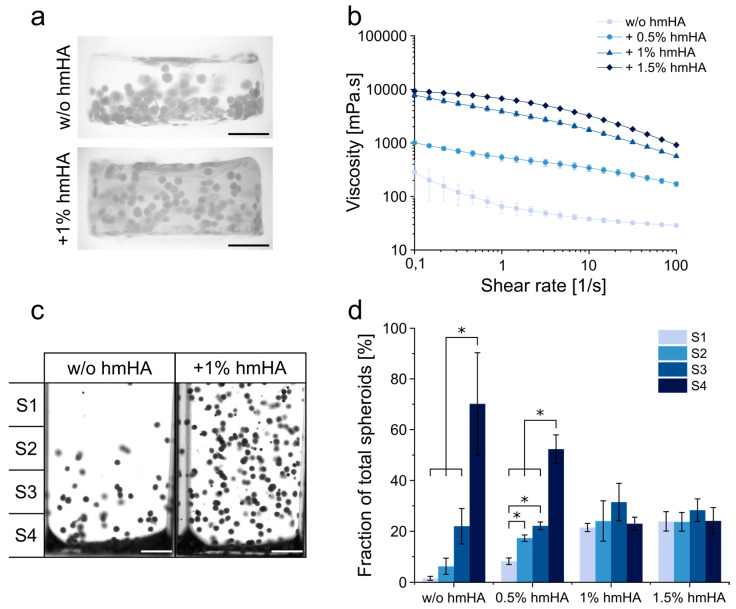
Modification of hyaluronic acid (HA) hydrogel formulation to ensure homogeneous spheroid distribution. (**a**) Representative images of printed gels without and with the addition of 1 wt% high molecular hyaluronic acid (hmHA). Scale bars represent 1000 µm. (**b**) Shear viscosity of the different hydrogel formulations without and with the addition of hmHA. Data are presented as mean ± standard deviation (*n* = 4). (**c**) Analysis of sedimentation behavior of human adipose-derived stromal cell (ASC) spheroids in non-crosslinked HA–SH hydrogel formulations without and with 1 wt% unmodified hmHA after 30 min. Images were divided into 4 sectors for further analysis (S1–S4). Scale bars represent 1000 µm. (**d**) Fraction of spheroids in sectors S1–S4 after 30 min in different gel formulations (without and with 0.5, 1 and 1.5 wt% hmHA). Data are presented as mean ± standard deviation (*n* = 3). Statistically significant differences are indicated by * (*p* < 0.05).

The printability of the ASC spheroids and the printing process’s influence on spheroid integrity, viability and distribution within the constructs were assessed in an extrusion-based printing setup. Spheroids were dispersed in a solution of thiol-modified hyaluronic acid (HA–SH), which was UV-crosslinked to stable hydrogels with allyl-modified poly(glycidol) (P(AGE-co-G), as previously shown in a similar form for bioprinting of mesenchymal stromal cells for cartilage engineering [36]. When using 0.8 wt% HA–SH with 0.5 wt% P(AGE-co-G) as the standard hydrogel formulation, spheroids tended to sediment during the printing and crosslinking process, which eventually led to an inhomogeneous dispersion of spheroids in the gels (Figure 2a). To prevent sedimentation and ensure homogeneous spheroid distribution, different amounts of unmodified high molecular hyaluronic acid (hmHA) were added to the gel formulation to enhance viscosity (Figure 2). Rheological characterization verified that the addition of 1 wt% and 1.5 wt% hmHA markedly increased the viscosity of the uncrosslinked gel precursor solution (Figure 2b). In a 30 min sedimentation assay, both 1 wt% and 1.5 wt% hmHA were observed to prevent sedimentation and resulted in a homogeneous dispersion of spheroids (Figure 2c,d). In all following bioprinting experiments, 1 wt% hmHA was added to the bioink formulation.

In extrusion-based bioprinting, fluid shear stress that increases with smaller needle diameter and increasing printing pressure may affect the viability of printed cells and impair the biological outcome, albeit this has so far only been shown for single cells [42,43,44,45]. Here, spheroids were printed with varying printing pressure and nozzle diameters, and their viability was assessed. Furthermore, to uncouple the printing process from construct geometry, printed and cast hydrogels were generated in identical shape (discs with 5 mm diameter and 2 mm height) using an extrusion-based printing setup as previously described [36], enabling comparability and avoiding differences in nutrient and oxygen supply associated with differently shaped constructs [36,46,47].

**Figure 3:**
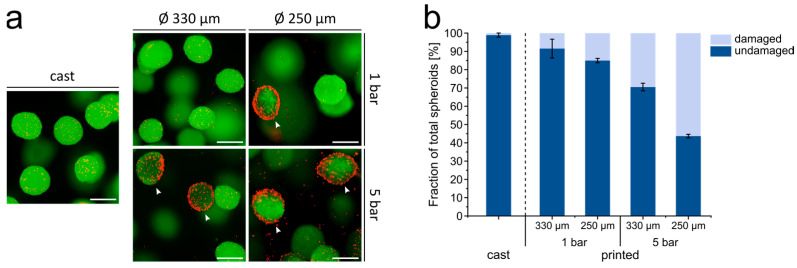
Influence of different printing parameters on the viability of ASC spheroids. (**a**) Live/dead staining of ASC spheroids either cast or printed with different printing pressure (1 and 5 bar) and different inner nozzle diameter (250 µm and 330 µm). Living cells are labeled green (calcein-AM), and dead cells are labeled red (EthD-III). White arrowheads indicate dead cells in the outer cell layer of the spheroids. Scale bars represent 200 µm. (**b**) Quantification of damaged and undamaged ASC spheroids after casting or printing under different conditions. Data are presented as mean ± standard deviation (*n* = 3 gels, with >80 spheroids each). All groups were statistically significantly different from each other (*p* < 0.05).

When printed with a nozzle with 330 µm inner diameter and 1 bar pressure, the percentage of spheroids with a noticeable fraction of dead cells at the outer, surface-exposed cell layer (in the following referred to as damaged spheroids) was 9%, in comparison to 1% for spheroids in the cast samples (Figure 3). With higher printing pressure (5 bar) and lower nozzle diameter (250 µm), the percentage of damaged spheroids increased dramatically up to 56% and large numbers of dead cells were observed at the outer region of the spheroids (Figure 3a,b). High pressure also led to abrasion of dead cells from the spheroid surface and their distribution in the surrounding gel, whereas cells in the spheroid core seemed to be more protected against these shear forces (Figure 3a). In all subsequent experiments, the printing of the ASC spheroids was done through a 330 µm nozzle at a pressure of 1 bar.

### 3.3. Adipogenesis in 3D Bioprinted ASC Spheroids

**Figure 4:**
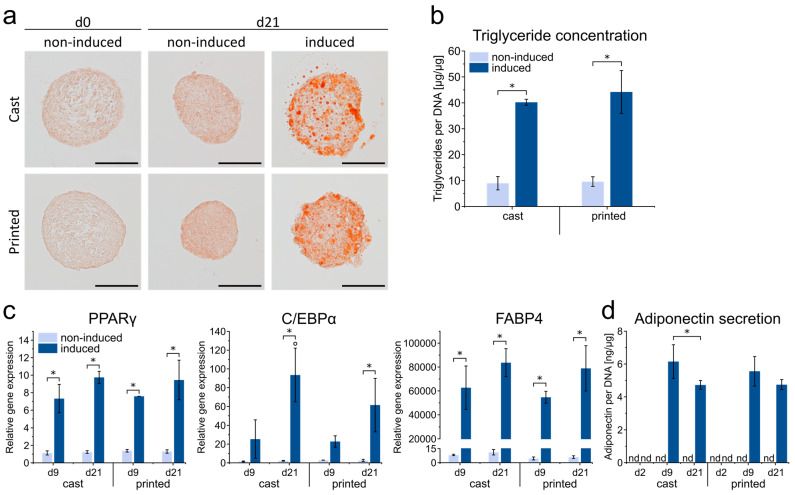
Adipogenic differentiation potential of ASC spheroids cast and printed in HA–SH/P(AGE-co-G) hydrogels. (**a**) Oil Red O staining of accumulated triglycerides in the cast and printed ASC spheroids after 21 days of adipogenic induction, as compared to non-induced controls (d0 and d21). Scale bars represent 100 µm. (**b**) Determination of triglyceride content after 21 days normalized to the DNA content of the respective samples. Data are presented as mean ± standard deviation (*n* = 3). Statistically significant differences are indicated by * (*p* < 0.05). (**c**) Adipogenic marker gene expression determined by qRT–PCR at days 0, 9 and 21. Gene expression was normalized to EF1α; obtained values were further normalized to day 0. Data are presented as mean ± standard deviation (*n* = 3). Statistically significant differences (*p* < 0.05) between induced and non-induced samples on the same day are indicated by * and between different days in the same group by °. (**d**) Adiponectin secretion as determined by enzyme-linked immunosorbent assay of the supernatants of cast vs. printed spheroids after 2, 9 and 21 days of adipogenic induction. Non-induced spheroids served as control. Obtained values were normalized to the DNA content of the respective samples. Data are presented as mean ± standard deviation (*n* = 3). Statistically significant differences are indicated by * (*p* < 0.05).

In order to induce adipose differentiation of the printed ASC spheroids, the spheroid-containing hydrogels were cultured in an adipogenic medium. To test whether the printing process may have impaired the differentiation of the spheroids, adipogenesis in printed and cast constructs was compared. After 21 days of adipogenic induction, spheroids in printed and cast constructs showed substantial adipogenic differentiation with lipid droplets in the spheroids clearly visible, as demonstrated by Oil Red O staining. This was in contrast to non-induced controls, where no lipid droplets were detectable (Figure 4a). Differentiated spheroids retained their rounded shape with some bulges at the surface due to embedded lipid droplets after cultivation for 21 days in an adipogenic medium. These findings were corroborated by quantitative analysis of the intracellular triglyceride content, which was distinctly higher in the induced samples compared to the non-induced controls, with comparable levels in the printed and cast samples (Figure 4b).

Gene expression analysis of PPARγ and C/EBPα, master regulators of early adipogenesis, as well as fatty acid-binding protein 4 (FABP4) as a late marker of adipogenic differentiation, also revealed no significant differences between the print and cast condition for all genes and time points analyzed (Figure 4c). Compared to non-induced controls, PPARγ and FABP4 displayed strongly elevated expression levels at d9 and d21 upon induction; for C/EBPα, increases in gene expression were significant at d21. In addition, secretion of adiponectin, a major adipokine, was examined by ELISA (Figure 4d). Adiponectin was not detectable in the supernatants of non-induced samples, whereas a marked increase in the secretion was observed in the induced samples at later stages of differentiation. Again, no significant differences were found in the secretion behavior between print and cast conditions at the respective time points. Taken together, ASC spheroids in the printed HA gel were demonstrated to be able to undergo substantial adipogenesis. Adipogenic differentiation was comparable in printed and cast constructs in terms of lipid storage, expression of adipogenic marker genes, and adipokine secretion, indicating that the printing process did not impair the differentiation behavior of the cells. With regard to the used hydrogel material, HA as part of the ECM of adipose tissue has been reported to act as a positive regulator of adipogenesis, making hyaluronan-based hydrogels suitable for the generation of 3D adipose constructs [48,49,50]. Our findings that the printing process does not impair the differentiation behavior of ASC spheroids are consistent with a previous study on the adipogenic differentiation of ASC spheroids printed in GelMA hydrogels [16].

**Figure 5:**
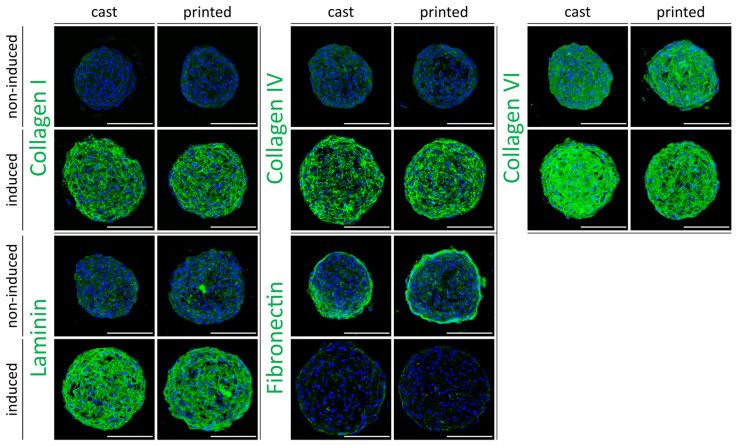
Immunohistochemical analysis of extracellular matrix (ECM) development in ASC spheroids cast and printed in HA–SH/P(AGE-co-G) hydrogels. Immunofluorescence staining of the major ECM components collagen I, collagen IV, collagen VI, laminin, and fibronectin in adipogenically induced vs. non-induced spheroids (day 9). Nuclei were counterstained with DAPI (blue). Scale bars represent 100 µm.

The development of a tissue-specific ECM is considered an important feature in 3D tissue constructs, especially for use in in vitro model systems, since, in addition to providing physical support, the ECM actively contributes to the behavior of the cells and the complex interaction with their environment [51]. We have recently shown that ECM composition in ASC spheroids that were adipogenically differentiated in culture plates closely resembled the ECM pattern in native adipose tissue [13]. As the encapsulation of the spheroids in the hydrogel and the printing process may affect ECM development, we evaluated the ECM deposition in printed versus cast spheroids. Furthermore, adipogenically differentiated spheroids were compared to non-induced controls. Constructs were stained immunohistochemically for the major ECM components collagen I, collagen IV, collagen VI, laminin, and fibronectin (Figure 5).

The ECM pattern of undifferentiated spheroids was characterized by a high expression of fibronectin and collagen VI, which is consistent with a stromal-like ECM phenotype. Adipose-specific constituents like collagen IV and laminin were barely detectable in these constructs. In contrast, adipogenically differentiated spheroids exhibited highly elevated expression of laminin and collagen IV, two main components of the adipocyte basement membrane. In addition, enhanced collagen I and collagen VI expression and a marked reduction of fibronectin were detected in the differentiated constructs in comparison to their undifferentiated counterparts (Figure 5). The observed ECM composition of the printed and adipogenically differentiated spheroids closely resembled the ECM composition of native adipose tissue, similar to our previous findings [13]. As demonstrated for the molecular markers during adipogenesis (see above), no visible differences in ECM deposition were observed between cast and printed constructs.

Taken together, the printing of ASC spheroids in a modified hyaluronic acid hydrogel and subsequent culture under adipogenic conditions allow us to generate adipose-like microtissues in printed constructs with sustained differentiation and tissue-specific ECM deposition.

### 3.4. Development of a Tumor-Adipose Tissue Co-Culture Model

**Figure 6:**
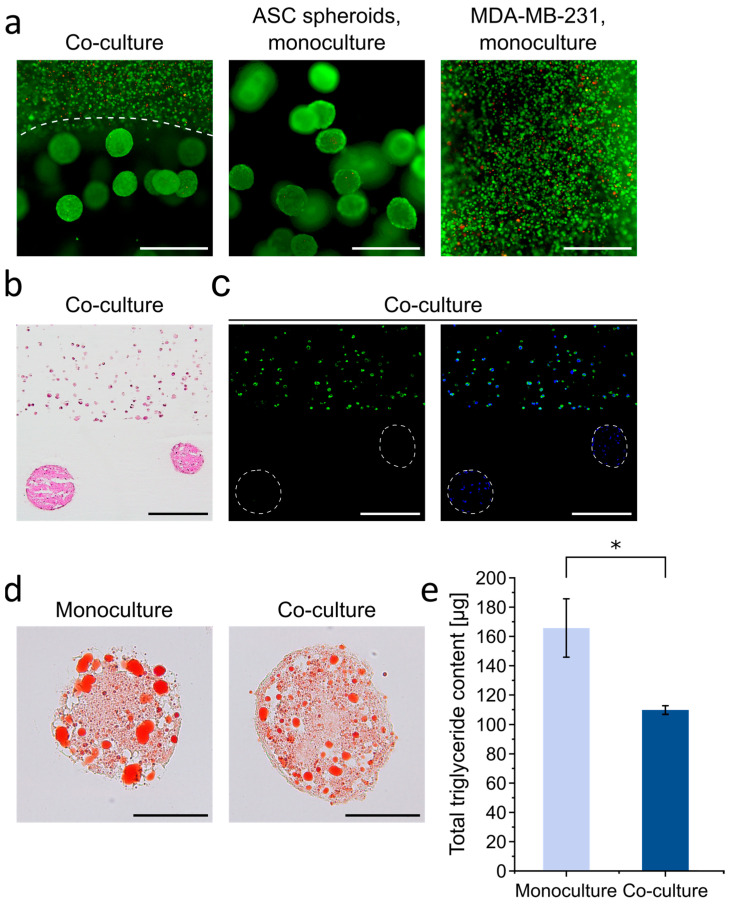
Printed breast cancer-adipose tissue model: viability, cell distribution and lipid content in adipose spheroids. ASC spheroids were printed in HA–SH/P(AGE-co-G) hydrogels, followed by adipogenic differentiation for 21 days. Subsequently, MDA-MB-231 breast cancer cells were printed on the adipose constructs using the same gel formulation, and co-culture was performed for 9 days (i.e., 30 days of total culture time). Constructs with differentiated ASC spheroids or MDA-MB-231 cells in monoculture served as controls. (**a**) Live/dead staining of the printed ASC spheroid/MDA-MB-231 co-culture and the respective monocultures at day 30 of culture (21 days adipogenic differentiation of the ASC spheroid-seeded hydrogels, followed by 9 days co-cultivation with the MDA-MB-231-seeded hydrogels). Living cells are depicted in green (calcein-AM), dead cells are depicted in red (EthD-III). Scale bars represent 500 µm. (**b**) Histological H&E staining of ASC spheroid/MDA-MB-231 co-culture (day 30, i.e., day 9 of co-cultivation). Scale bar represents 200 µm. (**c**) Immunohistochemical staining of MDA-MB-231 cells in co-culture using an anti-pan cytokeratin antibody (green, left). Nuclei were counterstained with DAPI (blue, right) (day 30, i.e., day 9 of co-cultivation). Scale bars represent 200 µm. (**d**) Visualization of accumulated triglycerides by Oil Red O staining of representative ASC spheroids in mono- or co-culture (day 30, i.e., day 9 of co-cultivation). Scale bars represent 100 µm. (**e**) Quantification of total triglyceride content of ASC spheroids in the ASC spheroid/MDA-MB-231 co-culture compared to monocultured spheroids (day 30, i.e., day 9 of co-cultivation). Data are presented as mean ± standard deviation (*n* = 3). Statistically significant differences are indicated by * (*p* < 0.05).

In the next step, we used the 3D adipose tissue constructs for a modular breast cancer model to facilitate investigations of the interaction between adipose tissue and breast cancer cells in a 3D context. Here, a particular focus was put on alterations in the adipose tissue. To this end, a tumor compartment consisting of an invasive tumor cell line (MDA-MB-231) was printed on top of the adipose tissue construct using the same HA-based hydrogel as bioink since HA is also a major ECM component of the breast cancer microenvironment [52,53,54]. In detail, ASC spheroids were printed and adipogenically differentiated for 21 days as described above. During this time, a limited shrinkage of the gel size (15–20%) occurred; however, the shape and the surface of the printed adipose construct remained virtually unaffected and allowed the printing of an even hydrogel layer on top. After 21 days, HA hydrogels containing the MDA-MB-231 cells were printed onto the matured adipose constructs. The resulting co-constructs were then cultured for a further nine days. Constructs with either only differentiated ASC spheroids or MDA-MB-231 single cells served as controls. ASC spheroids as well as tumor cells retained high viability after printing and in prolonged culture, as shown by live/dead staining (Figure 6a). This is in agreement with findings from a previous study where adipocytes and breast cancer cells were co-cultured in printed alginate/gelatin hydrogels [55]. As shown by histological H&E staining of cross-sections of the co-culture constructs and immunohistochemical staining of the cancer cells with an anti-pan cytokeratin antibody, the cancer cells remained in their compartment and did not migrate into the adipose compartment (Figure 6b,c).

Histological assessment of lipid storage by Oil Red O staining revealed a considerable reduction of lipid content in the adipose microtissues upon co-culture with MDA-MB-231 cells as compared with the monoculture control (Figure 6d). This observation was confirmed by quantitative measurements of intracellular triglycerides. In the co-culture model, levels of triglyceride content were significantly lower as compared to the monoculture, demonstrating a distinct effect of the tumor cells on the adipose compartment (Figure 6e). A similar reduction in lipid content of adipocytes by tumor cells has been described in native samples of breast tissue and also in in vitro studies, which has been interpreted as a cancer cell-induced reprogramming of adipocyte morphology and function [56,57,58,59]. The tumor-driven adipocyte dedifferentiation has been attributed to lipolysis and an alteration in canonical adipocyte-differentiating factors, although the precise mechanisms that may be involved in this process remain to be discovered [60].

Adipocytes in the vicinity of tumor cells have been shown to play a vital role in defining the ECM environment through enhanced secretion of factors, such as fibrillar collagens and fibronectin, which are associated with matrix stiffening and fibrosis [25]. The resulting fibrotic phenotype of the ECM is considered a key characteristic of breast cancer, but also of other tumors, and is associated with enhanced tumor aggressiveness [61,62].

To investigate whether the characteristic remodeling of the adipose ECM in the vicinity of breast cancer cells can be recapitulated in our printed breast cancer model, we examined the ECM pattern in the adipose microtissues upon co-cultivation.

**Figure 7:**
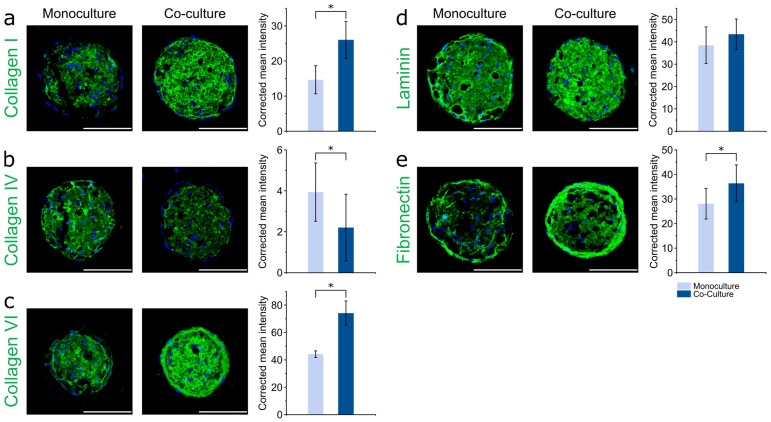
ECM deposition in adipose spheroids within the printed 3D co-culture model. ASC spheroids were printed in HA-SH/P(AGE-co-G) hydrogels, followed by adipogenic differentiation for 21 days. Subsequently, MDA-MB-231 cells were printed on the adipose constructs using the same gel formulation, and co-culture was performed for 9 days. Immunofluorescence staining of (**a**) collagen I, (**b**) collagen IV, (**c**) collagen VI, (**d**) laminin and (**e**) fibronectin, and quantification of the respective fluorescence intensities. Nuclei were counterstained with DAPI (blue). ASC spheroids in monoculture and co-culture on day 9 of the experiment are compared. Representative images are shown. Scale bars represent 100 µm. Data are presented as mean ± standard deviation (*n* = 10). Statistically significant differences are indicated by * (*p* < 0.05).

Adipose microtissues in co-culture were immunohistochemically stained for the major ECM components collagen I, IV, and VI, laminin, and fibronectin and compared to adipose constructs without tumor compartment after nine days. In the co-culture setup, adipose spheroids showed substantially increased expression of collagen I and VI and fibronectin (Figure 7a,c,e). Quantitative assessment of the respective ECM staining corroborated these findings. In contrast, collagen IV expression was significantly lower in the tumor model (Figure 7b), laminin expression was found to be similar (Figure 7d). An enhanced deposition of fibronectin and collagen I by cancer-associated adipocytes was previously reported in a murine transwell-based co-culture [63]. Fibronectin and collagen I are described to be key players in tumor progression, as their overexpression leads to increased matrix stiffness and concomitantly altered biochemical signaling and cell behavior [64]. Collagen VI has previously been shown to be abundantly expressed and secreted by adipocytes and to be involved in cancer cell–adipocyte heterotypic signaling. Increased stromal expression of collagen VI has been correlated with various aspects of tumor progression, such as proliferation, invasion and chemoresistance [65,66]. The observed ECM modifications in the adipose compartment of our model are also in agreement with data from in vivo studies that showed enhanced collagen VI expression in a mouse breast cancer model [61,62,66].

Altogether, we successfully demonstrated a distinct interaction between breast cancer cells and adipocytes in the printed co-culture. The results reflect phenomena observed in vivo, i.e., reduced lipid content and ECM remodeling, highlighting the relevance of the newly established model. In the absence of direct cell–cell contact, the observed alterations in the adipocyte phenotype are most likely mediated by the secretory activity of the tumor cells. Ongoing studies include investigations into the secretory function of adipocytes as well as tumor cells and the role of lipid metabolism and specific ECM components in tumor cell proliferation and migration, also using modified hydrogel formulations that support migration of the tumor cells. Furthermore, due to its modular design and the possible long-term culture, the presented model may also be utilized to investigate the interaction of ASC-enriched lipografts and breast cancer cells in the context of breast reconstruction in an in vivo-like 3D environment, which is an important aspect to evaluate this treatment in terms of oncological risk [67].

## 4. Conclusions

In this study, we first developed a printed 3D adipose tissue model based on ASC spheroids encapsulated in HA hydrogels and differentiated into adipose microtissues with an intrinsic tissue-specific ECM deposition. Printing process parameters for spheroids were evaluated and optimized, resulting in high viability and homogeneous dispersion of the spheroids in the printed constructs. Adipogenic differentiation, including ECM development, was comparable in printed and cast constructs, demonstrating that the printing process did not impair the cells’ differentiation behavior. These adipose tissue constructs were implemented in a printed 3D breast cancer model as a proof-of-concept study. Analysis of the lipid content and ECM deposition indicated that co-culture with breast cancer cells induced characteristic alterations of the adipocyte function and matrix composition, demonstrating the heterotypic interaction between breast cancer cells and adipose tissue. Further development of the biofabricated 3D model with advanced bioinks, the ability to spatially arrange tumor cells and adipocytes, and the inclusion of other cell types may facilitate to further decipher the complex cell-cell and cell-matrix crosstalk in breast cancer in a defined, native-like environment.

## Figures and Tables

**Table 1:** Primer sequences for qRT–PCR.

Primer	Sequence
EF1α forward	5′-GCCCATGTGTGTTGAGAGC-3′
EF1α reverse	5′-CCGCAACTGTCTGTCTCATATC-3′
PPARγ forward	5′-TTCAGAAATGCCTTGCAGTG-3′
PPARγ reverse	5′-CCAACAGCTTCTCCTTCTCG-3′
C/EBPα forward	5′-TGGACAAGAACAGCAACGAG-3′
C/EBPα reverse	5′-TTGTCACTGGTCAGCTCCAG-3′
FABP4 forward	5′-CATACTGGGCCAGGAATTTG-3′
FABP4 reverse	5′-TACCAGGACACCCCCATCTA-3′

## Data Availability

Data is contained within the article.
